# Supplementary material for: Ranking of critical species to preserve the functionality of mutualistic networks using the k-core decomposition
Source: PeerJ. 2017 May 18;5:e3321. doi: 10.7717/peerj.3321 (PMC5438587; doi:10.7717/peerj.3321)
Supplement: Supplemental Information 1 [file peerj-05-3321-s001.doc]

**Table S1.** Average number of removed species to destroy half the Giant Component, according to the different indexes: k_risk_ is the top performer for 67 networks, degree for 48, k_degree_ for 39 and eigenvector centrality for 28 networks.

| **Network** | **GC size** | **k_risk_** | **degree** | **k_degree_** | **eigenc** |  | **Network** | **GC size** | **k_risk_** | **degree** | **k_degree_** | **eigenc** |
| --- | --- | --- | --- | --- | --- | --- | --- | --- | --- | --- | --- | --- |
| PL_001 | 177 | 21.73 | 22.13 | 22 | 23 |  | PL_045 | 41 | 6 | 5.45 | 7 | 8 |
| PL_002 | 103 | 14.46 | 12.51 | 13 | 15 |  | PL_046 | 60 | 11 | 11.94 | 13 | 14 |
| PL_003 | 61 | 5 | 5.35 | 6 | 6 |  | PL_047 | 205 | 4 | 4 | 4 | 4 |
| PL_004 | 112 | 3 | 3 | 3 | 3 |  | PL_048 | 266 | 10 | 10 | 9 | 12 |
| PL_005 | 361 | 25 | 30.73 | 36 | 42 |  | PL_049 | 262 | 11 | 13 | 15 | 16 |
| PL_006 | 78 | 3 | 3 | 3 | 3 |  | PL_050 | 49 | 6 | 6.36 | 7 | 7 |
| PL_007 | 50 | 5 | 4 | 4 | 4 |  | PL_051 | 104 | 3 | 3 | 3 | 3 |
| PL_008 | 49 | 6 | 7 | 7 | 11 |  | PL_052 | 52 | 6 | 6 | 6 | 7 |
| PL_009 | 142 | 7 | 7.52 | 8 | 12 |  | PL_053 | 364 | 19 | 22.49 | 23 | 34 |
| PL_010 | 107 | 23.46 | 29 | 29 | 32 |  | PL_054 | 414 | 23 | 25.23 | 27 | 30 |
| PL_011 | 27 | 4 | 5.04 | 6 | 6 |  | PL_055 | 253 | 16 | 16.4 | 17 | 19 |
| PL_012 | 84 | 7 | 7 | 7 | 7 |  | PL_056 | 456 | 22 | 28.71 | 33 | 43 |
| PL_013 | 65 | 4 | 4 | 4 | 5 |  | PL_057 | 997 | 17 | 17 | 20 | 36 |
| PL_014 | 108 | 6 | 5 | 5 | 6 |  | PL_058 | 111 | 14 | 17.55 | 19 | 20 |
| PL_015 | 793 | 48 | 56 | 60 | 87 |  | PL_059 | 26 | 6 | 5 | 5 | 5 |
| PL_016 | 205 | 9 | 9 | 10 | 17 |  | SD_001 | 28 | 3 | 3 | 3 | 5 |
| PL_017 | 104 | 9 | 10.52 | 11 | 11 |  | SD_002 | 40 | 5 | 5 | 5 | 5 |
| PL_018 | 144 | 18 | 19 | 23 | 24 |  | SD_003 | 41 | 4 | 4 | 4 | 4 |
| PL_019 | 123 | 14 | 15.37 | 16 | 18 |  | SD_004 | 52 | 4 | 4 | 4 | 4 |
| PL_020 | 109 | 3 | 3 | 3 | 3 |  | SD_005 | 34 | 3 | 3 | 3 | 3 |
| PL_021 | 766 | 12 | 12 | 12 | 38 |  | SD_006 | 34 | 4 | 4.31 | 5 | 5 |
| PL_022 | 66 | 4 | 4 | 4 | 4 |  | SD_007 | 79 | 3 | 3 | 3 | 3 |
| PL_023 | 90 | 3 | 3 | 3 | 3 |  | SD_008 | 26 | 9.83 | 8.45 | 8 | 11 |
| PL_024 | 22 | 4 | 3.55 | 3 | 3 |  | SD_009 | 25 | 3 | 4 | 4 | 5 |
| PL_025 | 57 | 6 | 6 | 6 | 10 |  | SD_010 | 64 | 8 | 8 | 9 | 13 |
| PL_026 | 150 | 2 | 2 | 2 | 2 |  | SD_011 | 25 | 6 | 5.33 | 5 | 6 |
| PL_027 | 75 | 8.54 | 8 | 9 | 11 |  | SD_012 | 64 | 12.71 | 12.53 | 12 | 14 |
| PL_028 | 180 | 13 | 14 | 16 | 24 |  | SD_013 | 55 | 11 | 8 | 19 | 14 |
| PL_029 | 167 | 17 | 16.97 | 17 | 19 |  | SD_014 | 33 | 9 | 10 | 10 | 10 |
| PL_030 | 70 | 10.23 | 6.65 | 7 | 13 |  | SD_015 | 32 | 4 | 4 | 4 | 4 |
| PL_031 | 91 | 9.53 | 7.92 | 13 | 17 |  | SD_016 | 85 | 17 | 18 | 20 | 23 |
| PL_032 | 40 | 2 | 2 | 2 | 2 |  | SD_017 | 24 | 7.28 | 6.62 | 10 | 10 |
| PL_033 | 47 | 8.65 | 8 | 10 | 12 |  | SD_018 | 53 | 4.24 | 5 | 5 | 5 |
| PL_034 | 151 | 6 | 7.54 | 8 | 9 |  | SD_019 | 209 | 13 | 16.48 | 20 | 21 |
| PL_035 | 97 | 9 | 8.93 | 9 | 9 |  | SD_020 | 58 | 7.65 | 9.22 | 10 | 10 |
| PL_036 | 22 | 3.68 | 2 | 2 | 2 |  | SD_021 | 46 | 9 | 10 | 10 | 10 |
| PL_037 | 50 | 5 | 5 | 5 | 7 |  | SD_022 | 317 | 39 | 50.43 | 53 | 60 |
| PL_038 | 50 | 4.36 | 4 | 4 | 7 |  | SD_023 | 23 | 4 | 4 | 4 | 4 |
| PL_039 | 68 | 6 | 6.68 | 9 | 10 |  | SD_024 | 19 | 5.88 | 6 | 7 | 8 |
| PL_040 | 70 | 8.56 | 7 | 9 | 10 |  | SD_025 | 13 | 4.38 | 4.16 | 4.16 | 4 |
| PL_041 | 70 | 10 | 10 | 11 | 12 |  | SD_026 | 6 | 2 | 2 | 2 | 2 |
| PL_042 | 16 | 2 | 2 | 2 | 2 |  | SD_027 | 16 | 3 | 3 | 3 | 3 |
| PL_043 | 110 | 12 | 14 | 14 | 19 |  | SD_028 | 13 | 3 | 3 | 3 | 3 |
| PL_044 | 712 | 21 | 23 | 25 | 49 |  | SD_029 | 9 | 2 | 2 | 2 | 2 |
|  |  |  |  |  |  |  | SD_030 | 9 | 2 | 2.65 | 2 | 2 |

**Table S2.** Average area under the extinction curve, when the surviving fraction of plant species is measured. *MusRank* is the top performer for 85 networks, k_risk_ for 9, degree for 8, k_degree_ for 7 and eigenvector centrality for 7.

| **Network** | **MusRank** | **k_risk_** | **k_degree_** | **degree** | **eigenc** |  | **Network** | **MusRank** | **k_risk_** | **k_degree_** | **degree** | **eigenc** |
| --- | --- | --- | --- | --- | --- | --- | --- | --- | --- | --- | --- | --- |
| PL_001 | 0.3121 | 0.4236 | 0.4115 | 0.3956 | 0.4535 |  | PL_045 | 0.3324 | 0.4052 | 0.4047 | 0.4116 | 0.4063 |
| PL_002 | 0.3588 | 0.4710 | 0.4641 | 0.4555 | 0.4856 |  | PL_046 | 0.6577 | 0.7308 | 0.7528 | 0.7445 | 0.7642 |
| PL_003 | 0.2896 | 0.3505 | 0.3085 | 0.3232 | 0.3563 |  | PL_047 | 0.3091 | 0.6283 | 0.6541 | 0.6302 | 0.7143 |
| PL_004 | 0.2821 | 0.6579 | 0.6695 | 0.6536 | 0.7900 |  | PL_048 | 0.3336 | 0.6334 | 0.6594 | 0.6471 | 0.6887 |
| PL_005 | 0.2755 | 0.4836 | 0.5198 | 0.4906 | 0.5573 |  | PL_049 | 0.3220 | 0.7150 | 0.6773 | 0.6880 | 0.7757 |
| PL_006 | 0.2490 | 0.4520 | 0.4882 | 0.4344 | 0.5420 |  | PL_050 | 0.3976 | 0.5635 | 0.5114 | 0.4815 | 0.5918 |
| PL_007 | 0.3294 | 0.4982 | 0.4920 | 0.4702 | 0.5769 |  | PL_051 | 0.3115 | 0.6132 | 0.7000 | 0.5870 | 0.7008 |
| PL_008 | 0.5251 | 0.7287 | 0.7093 | 0.7117 | 0.7213 |  | PL_052 | 0.4121 | 0.6289 | 0.6514 | 0.6132 | 0.6761 |
| PL_009 | 0.3583 | 0.6689 | 0.6315 | 0.6473 | 0.6857 |  | PL_053 | 0.2510 | 0.5253 | 0.4590 | 0.5081 | 0.5058 |
| PL_010 | 0.6004 | 0.6948 | 0.7044 | 0.7003 | 0.7150 |  | PL_054 | 0.2568 | 0.5035 | 0.5136 | 0.4885 | 0.5835 |
| PL_011 | 0.3876 | 0.4272 | 0.3994 | 0.3999 | 0.4290 |  | PL_055 | 0.2929 | 0.5548 | 0.5795 | 0.5633 | 0.6378 |
| PL_012 | 0.2716 | 0.3860 | 0.3417 | 0.3494 | 0.3604 |  | PL_056 | 0.2696 | 0.5717 | 0.5545 | 0.5605 | 0.5885 |
| PL_013 | 0.4353 | 0.8088 | 0.7716 | 0.7606 | 0.7212 |  | PL_057 | 0.2019 | 0.5392 | 0.5217 | 0.5261 | 0.5576 |
| PL_014 | 0.2726 | 0.4935 | 0.5617 | 0.4840 | 0.6980 |  | PL_058 | 0.4168 | 0.5507 | 0.5585 | 0.5683 | 0.5773 |
| PL_015 | 0.3792 | 0.6637 | 0.6579 | 0.6633 | 0.6859 |  | PL_059 | 0.3639 | 0.3587 | 0.3649 | 0.3631 | 0.3757 |
| PL_016 | 0.3641 | 0.7275 | 0.6973 | 0.6719 | 0.7108 |  | SD_001 | 0.4592 | 0.5459 | 0.5141 | 0.5229 | 0.5068 |
| PL_017 | 0.3703 | 0.4895 | 0.5187 | 0.4886 | 0.5532 |  | SD_002 | 0.5000 | 0.4911 | 0.5000 | 0.4919 | 0.5000 |
| PL_018 | 0.4451 | 0.6194 | 0.6249 | 0.6259 | 0.6504 |  | SD_003 | 0.3051 | 0.3281 | 0.3166 | 0.3059 | 0.3166 |
| PL_019 | 0.3474 | 0.5391 | 0.5373 | 0.5320 | 0.5658 |  | SD_004 | 0.2121 | 0.2351 | 0.2389 | 0.2350 | 0.2476 |
| PL_020 | 0.2674 | 0.5480 | 0.5427 | 0.5199 | 0.6005 |  | SD_005 | 0.2656 | 0.3587 | 0.3546 | 0.2718 | 0.3974 |
| PL_021 | 0.1761 | 0.5147 | 0.5092 | 0.4985 | 0.6207 |  | SD_006 | 0.3078 | 0.3515 | 0.3471 | 0.3444 | 0.3588 |
| PL_022 | 0.2511 | 0.5003 | 0.5019 | 0.4304 | 0.7444 |  | SD_007 | 0.2528 | 0.2528 | 0.2528 | 0.2528 | 0.2528 |
| PL_023 | 0.2285 | 0.4959 | 0.7041 | 0.4517 | 0.8068 |  | SD_008 | 0.6875 | 0.6861 | 0.7125 | 0.7108 | 0.7188 |
| PL_024 | 0.4111 | 0.5650 | 0.5449 | 0.5283 | 0.5444 |  | SD_009 | 0.4167 | 0.5395 | 0.5033 | 0.5453 | 0.6389 |
| PL_025 | 0.4344 | 0.5956 | 0.6641 | 0.6030 | 0.6792 |  | SD_010 | 0.4929 | 0.4986 | 0.5271 | 0.5129 | 0.5314 |
| PL_026 | 0.2138 | 0.3362 | 0.3811 | 0.2874 | 0.4052 |  | SD_011 | 0.5286 | 0.5927 | 0.5422 | 0.5623 | 0.5422 |
| PL_027 | 0.4466 | 0.6711 | 0.6179 | 0.6728 | 0.6185 |  | SD_012 | 0.3912 | 0.4140 | 0.4321 | 0.4316 | 0.4332 |
| PL_028 | 0.3266 | 0.5613 | 0.6178 | 0.5754 | 0.6621 |  | SD_013 | 0.4835 | 0.5629 | 0.6754 | 0.5885 | 0.6462 |
| PL_029 | 0.3107 | 0.5061 | 0.4865 | 0.4919 | 0.6198 |  | SD_014 | 0.5221 | 0.5504 | 0.5415 | 0.5441 | 0.5404 |
| PL_030 | 0.3949 | 0.6113 | 0.6054 | 0.5870 | 0.6329 |  | SD_015 | 0.7444 | 0.8602 | 0.8633 | 0.8607 | 0.8556 |
| PL_031 | 0.3314 | 0.4078 | 0.3857 | 0.3926 | 0.4128 |  | SD_016 | 0.6318 | 0.6812 | 0.6830 | 0.6816 | 0.6872 |
| PL_032 | 0.3889 | 0.5157 | 0.6312 | 0.4995 | 0.6255 |  | SD_017 | 0.6406 | 0.6719 | 0.6875 | 0.6639 | 0.6875 |
| PL_033 | 0.6640 | 0.6732 | 0.6753 | 0.6832 | 0.6799 |  | SD_018 | 0.3413 | 0.5645 | 0.4809 | 0.4596 | 0.5361 |
| PL_034 | 0.2497 | 0.4697 | 0.4383 | 0.4563 | 0.5204 |  | SD_019 | 0.3185 | 0.3444 | 0.3602 | 0.3535 | 0.3849 |
| PL_035 | 0.3121 | 0.3648 | 0.3886 | 0.3633 | 0.4085 |  | SD_020 | 0.3155 | 0.3459 | 0.3608 | 0.3485 | 0.3722 |
| PL_036 | 0.4306 | 0.4677 | 0.4667 | 0.4560 | 0.4750 |  | SD_021 | 0.4254 | 0.4579 | 0.4682 | 0.4567 | 0.4737 |
| PL_037 | 0.5153 | 0.7303 | 0.6521 | 0.6924 | 0.6400 |  | SD_022 | 0.3517 | 0.3720 | 0.3886 | 0.3820 | 0.4284 |
| PL_038 | 0.4702 | 0.7328 | 0.6948 | 0.7310 | 0.7411 |  | SD_023 | 0.3875 | 0.4000 | 0.3875 | 0.3875 | 0.3875 |
| PL_039 | 0.3454 | 0.5556 | 0.4979 | 0.5425 | 0.5046 |  | SD_024 | 0.5130 | 0.5542 | 0.5357 | 0.5114 | 0.5357 |
| PL_040 | 0.2940 | 0.4478 | 0.4696 | 0.4153 | 0.6227 |  | SD_025 | 0.4722 | 0.5692 | 0.5556 | 0.5156 | 0.5556 |
| PL_041 | 0.3978 | 0.5253 | 0.5562 | 0.5238 | 0.5566 |  | SD_026 | 0.3333 | 0.3333 | 0.3333 | 0.3333 | 0.3333 |
| PL_042 | 0.3452 | 0.3621 | 0.3690 | 0.3621 | 0.3690 |  | SD_027 | 0.4750 | 0.4750 | 0.4750 | 0.4750 | 0.4750 |
| PL_043 | 0.4414 | 0.6364 | 0.6113 | 0.6183 | 0.6430 |  | SD_028 | 0.4429 | 0.4429 | 0.4429 | 0.4429 | 0.4429 |
| PL_044 | 0.2401 | 0.5932 | 0.5701 | 0.5723 | 0.6721 |  | SD_029 | 0.5000 | 0.5000 | 0.5000 | 0.5000 | 0.5000 |
|  |  |  |  |  |  |  | SD_030 | 0.4583 | 0.4583 | 0.4583 | 0.4583 | 0.4583 |

**Table S3.** Average area under the extinction curve, when the surviving fraction of the original giant component is measured. The top performer is k_degree_ for 42 networks, degree for 24, k_risk_ for 21, eigenvector centrality for 18 and MusRank for 16.

| **Network** | **MusRank** | **k_risk_** | **k_degree_** | **degree** | **eigenc** |  | **Network** | **MusRank** | **k_risk_** | **k_degree_** | **degree** | **eigenc** |
| --- | --- | --- | --- | --- | --- | --- | --- | --- | --- | --- | --- | --- |
| PL_001 | 0.3158 | 0.2410 | 0.2212 | 0.2224 | 0.2546 |  | PL_045 | 0.4246 | 0.3052 | 0.3544 | 0.3066 | 0.3496 |
| PL_002 | 0.4337 | 0.3441 | 0.3244 | 0.3237 | 0.3198 |  | PL_046 | 0.5060 | 0.4975 | 0.5013 | 0.5008 | 0.4987 |
| PL_003 | 0.2176 | 0.2607 | 0.2093 | 0.2056 | 0.2145 |  | PL_047 | 0.4626 | 0.3694 | 0.3686 | 0.3694 | 0.3622 |
| PL_004 | 0.4661 | 0.3700 | 0.3744 | 0.3697 | 0.3843 |  | PL_048 | 0.4715 | 0.3895 | 0.3920 | 0.3909 | 0.3998 |
| PL_005 | 0.4380 | 0.2954 | 0.2935 | 0.2968 | 0.3482 |  | PL_049 | 0.4662 | 0.3350 | 0.3286 | 0.3372 | 0.3960 |
| PL_006 | 0.4374 | 0.4044 | 0.3929 | 0.4038 | 0.3932 |  | PL_050 | 0.4068 | 0.3641 | 0.3285 | 0.3387 | 0.3214 |
| PL_007 | 0.4465 | 0.3539 | 0.3233 | 0.3310 | 0.3513 |  | PL_051 | 0.4380 | 0.3428 | 0.3322 | 0.3384 | 0.3762 |
| PL_008 | 0.4786 | 0.4363 | 0.4370 | 0.4344 | 0.4386 |  | PL_052 | 0.4502 | 0.3228 | 0.3029 | 0.3227 | 0.3381 |
| PL_009 | 0.4331 | 0.2613 | 0.2703 | 0.2617 | 0.3195 |  | PL_053 | 0.4046 | 0.2018 | 0.2045 | 0.1980 | 0.2153 |
| PL_010 | 0.5086 | 0.4789 | 0.4783 | 0.4802 | 0.4974 |  | PL_054 | 0.4337 | 0.2427 | 0.2273 | 0.2481 | 0.3287 |
| PL_011 | 0.4169 | 0.3994 | 0.4108 | 0.3874 | 0.3985 |  | PL_055 | 0.4443 | 0.2602 | 0.2449 | 0.2443 | 0.3557 |
| PL_012 | 0.3451 | 0.2859 | 0.3030 | 0.2768 | 0.3173 |  | PL_056 | 0.4142 | 0.2451 | 0.2364 | 0.2460 | 0.3498 |
| PL_013 | 0.4718 | 0.3548 | 0.3302 | 0.3527 | 0.3828 |  | PL_057 | 0.4597 | 0.2378 | 0.2498 | 0.2377 | 0.3355 |
| PL_014 | 0.4378 | 0.3928 | 0.3697 | 0.3965 | 0.4679 |  | PL_058 | 0.4301 | 0.3578 | 0.3694 | 0.3622 | 0.3840 |
| PL_015 | 0.4780 | 0.4030 | 0.3991 | 0.4025 | 0.4177 |  | PL_059 | 0.4077 | 0.3916 | 0.3944 | 0.3934 | 0.3954 |
| PL_016 | 0.4689 | 0.3781 | 0.3221 | 0.3743 | 0.3946 |  | SD_001 | 0.4489 | 0.3792 | 0.3871 | 0.3732 | 0.3854 |
| PL_017 | 0.4579 | 0.4240 | 0.4190 | 0.4257 | 0.4234 |  | SD_002 | 0.4905 | 0.4849 | 0.4905 | 0.4847 | 0.4905 |
| PL_018 | 0.4498 | 0.3419 | 0.3421 | 0.3432 | 0.3585 |  | SD_003 | 0.3333 | 0.3222 | 0.2829 | 0.2999 | 0.2829 |
| PL_019 | 0.4441 | 0.3138 | 0.3083 | 0.3118 | 0.3265 |  | SD_004 | 0.2510 | 0.2286 | 0.2298 | 0.2288 | 0.2448 |
| PL_020 | 0.4433 | 0.3688 | 0.3426 | 0.3674 | 0.3894 |  | SD_005 | 0.3646 | 0.2592 | 0.2640 | 0.2762 | 0.2569 |
| PL_021 | 0.4563 | 0.2512 | 0.2136 | 0.2510 | 0.4071 |  | SD_006 | 0.3444 | 0.3072 | 0.3049 | 0.3088 | 0.3025 |
| PL_022 | 0.4184 | 0.3475 | 0.2828 | 0.3068 | 0.3858 |  | SD_007 | 0.2433 | 0.2433 | 0.2433 | 0.2433 | 0.2433 |
| PL_023 | 0.4327 | 0.3187 | 0.2840 | 0.3185 | 0.2901 |  | SD_008 | 0.6060 | 0.6056 | 0.6220 | 0.6221 | 0.6260 |
| PL_024 | 0.5028 | 0.3380 | 0.3406 | 0.3135 | 0.3389 |  | SD_009 | 0.3935 | 0.3280 | 0.3415 | 0.3296 | 0.3009 |
| PL_025 | 0.4529 | 0.4451 | 0.4521 | 0.4472 | 0.4582 |  | SD_010 | 0.4881 | 0.4926 | 0.5153 | 0.5040 | 0.5187 |
| PL_026 | 0.3245 | 0.2316 | 0.2153 | 0.2300 | 0.1976 |  | SD_011 | 0.4736 | 0.4678 | 0.4405 | 0.4452 | 0.4405 |
| PL_027 | 0.4590 | 0.2669 | 0.2676 | 0.2698 | 0.2912 |  | SD_012 | 0.3603 | 0.3291 | 0.3575 | 0.3429 | 0.3609 |
| PL_028 | 0.4445 | 0.3505 | 0.3444 | 0.3483 | 0.3743 |  | SD_013 | 0.4772 | 0.5185 | 0.6053 | 0.5466 | 0.5897 |
| PL_029 | 0.4270 | 0.3084 | 0.3024 | 0.3040 | 0.3272 |  | SD_014 | 0.4982 | 0.5117 | 0.5081 | 0.5092 | 0.5074 |
| PL_030 | 0.4658 | 0.3410 | 0.2504 | 0.2685 | 0.3469 |  | SD_015 | 0.5251 | 0.5392 | 0.5288 | 0.5397 | 0.5299 |
| PL_031 | 0.3190 | 0.2263 | 0.2603 | 0.2178 | 0.2866 |  | SD_016 | 0.4949 | 0.4832 | 0.4849 | 0.4833 | 0.4852 |
| PL_032 | 0.4322 | 0.3853 | 0.3810 | 0.3831 | 0.3951 |  | SD_017 | 0.5842 | 0.5734 | 0.5842 | 0.5832 | 0.5842 |
| PL_033 | 0.5326 | 0.4953 | 0.4910 | 0.4914 | 0.5160 |  | SD_018 | 0.4112 | 0.2159 | 0.1678 | 0.1806 | 0.2231 |
| PL_034 | 0.4489 | 0.3585 | 0.3596 | 0.3497 | 0.3664 |  | SD_019 | 0.3528 | 0.3729 | 0.3861 | 0.3807 | 0.4058 |
| PL_035 | 0.3636 | 0.3488 | 0.3498 | 0.3259 | 0.3595 |  | SD_020 | 0.4135 | 0.4156 | 0.4236 | 0.4169 | 0.4229 |
| PL_036 | 0.3708 | 0.3377 | 0.2785 | 0.2859 | 0.2917 |  | SD_021 | 0.4594 | 0.4617 | 0.4627 | 0.4610 | 0.4627 |
| PL_037 | 0.4010 | 0.2716 | 0.2652 | 0.2585 | 0.2997 |  | SD_022 | 0.3681 | 0.3525 | 0.3508 | 0.3522 | 0.3814 |
| PL_038 | 0.4366 | 0.3665 | 0.3767 | 0.3605 | 0.4366 |  | SD_023 | 0.3860 | 0.3934 | 0.3860 | 0.3860 | 0.3860 |
| PL_039 | 0.4274 | 0.3036 | 0.3042 | 0.3080 | 0.3083 |  | SD_024 | 0.4916 | 0.5098 | 0.5079 | 0.4931 | 0.5079 |
| PL_040 | 0.4042 | 0.3454 | 0.3037 | 0.3096 | 0.4164 |  | SD_025 | 0.4621 | 0.4702 | 0.4621 | 0.4732 | 0.4621 |
| PL_041 | 0.4394 | 0.3216 | 0.3344 | 0.3205 | 0.3550 |  | SD_026 | 0.4167 | 0.4167 | 0.4167 | 0.4167 | 0.4167 |
| PL_042 | 0.3833 | 0.3227 | 0.3167 | 0.3218 | 0.3167 |  | SD_027 | 0.4712 | 0.4712 | 0.4712 | 0.4712 | 0.4712 |
| PL_043 | 0.4577 | 0.3634 | 0.3558 | 0.3616 | 0.3959 |  | SD_028 | 0.4455 | 0.4455 | 0.4455 | 0.4455 | 0.4455 |
| PL_044 | 0.4410 | 0.2286 | 0.2058 | 0.2255 | 0.3669 |  | SD_029 | 0.4667 | 0.4667 | 0.4667 | 0.4667 | 0.4667 |
|  |  |  |  |  |  |  | SD_030 | 0.4583 | 0.4583 | 0.4583 | 0.4583 | 0.4583 |
